# Invasion of *Ureaplasma diversum *in Hep-2 cells

**DOI:** 10.1186/1471-2180-10-83

**Published:** 2010-03-17

**Authors:** Lucas Miranda Marques, Priscilla M Ueno, Melissa Buzinhani, Beatriz A Cortez, Renata L Neto, Maurício Yamaguti, Rosângela C Oliveira, Ana Márcia S Guimarães, Telma A Monezi, Antonio Carlos R Braga, Gláucia M Machado-Santelli, Jorge Timenetsky

**Affiliations:** 1 Departamento de Microbiologia, Instituto de Ciências Biomédicas, Universidade de São Paulo. Av. Professor Lineu Prestes, 1374. 05508-900, São Paulo, SP, Brazil; 2 Núcleo de Tecnologia em Saúde, Instituto Multidisciplinar em Saúde, Universidade Federal da Bahia. Av. Olívia Flores, 3000. 45055-090, Vitória da Conquista, BA, Brazil; 3 Departamento de Biologia Celular e do Desenvolvimento, Instituto de Ciências Biomédicas, Universidade de São Paulo. Av. Professor Lineu Prestes, 1374. CEP 05508 900, São Paulo, SP, Brazil

## Abstract

**Background:**

Understanding mollicutes is challenging due to their variety and relationship with host cells. Invasion has explained issues related to their opportunistic role. Few studies have been done on the *Ureaplasma diversum *mollicute, which is detected in healthy or diseased bovine. The invasion in Hep-2 cells of four clinical isolates and two reference strains of their ureaplasma was studied by Confocal Laser Scanning Microscopy and gentamicin invasion assay.

**Results:**

The isolates and strains used were detected inside the cells after infection of one minute without difference in the arrangement for adhesion and invasion. The adhesion was scattered throughout the cells, and after three hours, the invasion of the ureaplasmas surrounded the nuclear region but were not observed inside the nuclei. The gentamicin invasion assay detected that 1% of the ATCC strains were inside the infected Hep-2 cells in contrast to 10% to the clinical isolates. A high level of phospholipase C activity was also detected in all studied ureaplasma.

**Conclusions:**

The results presented herein will help better understand *U. diversum *infections, aswell as cellular attachment and virulence.

## Background

In 1956, mycoplasma and cell cultures were first associated in laboratory contamination [1]. This contamination affects research by invalidating results in diagnosis. However interference by these bacteria in mammalian non phagocytic cell cultures has been used to study mollicute biology [2].

The opportunism of *Mollicutes *is a challenging subject. These microbes are diverse enough to explain their relationship variety with the host cells [3]. The adhesion seems crucial for their pathogenicity [4]. In addition, some mollicutes have been detected inside non naturally phagocytic cells. In fact, the intracellular location is well protected from the immune system and some antibiotics [3]. The use of non-phagocytic cells to study mollicutes has been of great interest mainly since *Mycoplasma fermentans *was initially considered a cofactor in the pathogenesis of AIDS [5]. Other mycoplasmas showed this same characteristic when inoculated in non-phagocytic cells such as *M. fermentans *[6], *M. pneumoniae *[7], *M. genitalium *[8] and *M. gallisepticum *[9].

*Ureaplasma diversum *is a bovine-originated mollicute, first isolated in 1969 and considered a non-pathogenic species. Although detected in healthy animals, it is currently considered a pathogenic species due to its strong association with cattle diseases such as placentitis, fetal alveolitis, abortion and birth of weak calves [10]. As with most animal mycoplasmosis, the cause of Ureaplasma-associated reproductive disease is multifactorial [11]. In bulls, this ureaplasma is an important pathogen of the genital tract, involved in such diseases as lowered sperm motility, seminal vesiculitis, and epididymitis [12]. Nevertheless, little is known about the virulence and pathogenic mechanisms of this mollicute.

Because the invasion of *U. diversum *in not known, we inoculated this mollicute in Hep-2 cells and observed this infection through Confocal Laser Scanning Microscopy (CLSM) and used a gentamicin invasion assay.

## Results

### *U. diversum *adhesion and invasion on Hep-2 cells observed by CLSM

**Figure 1:**
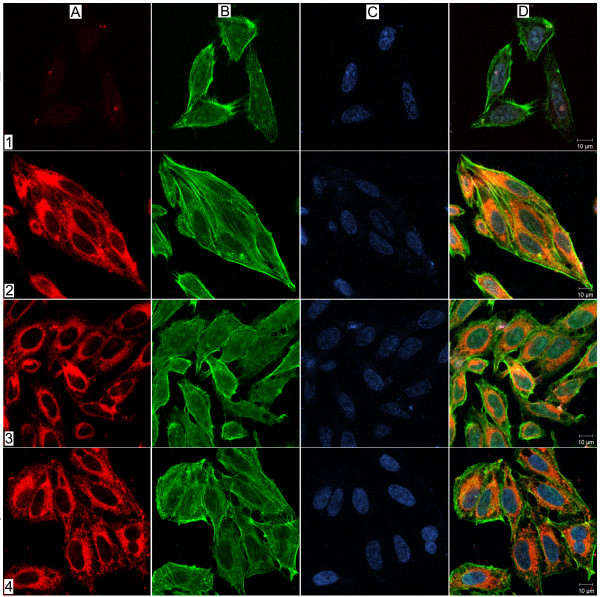
**Infection of *U. diversum *in HEp-2 cells**. LSCM optical sections showing internalization of *U. diversum *in HEp-2 cells after 1 minute (1), 30 minutes (2), 3 hours (3) and 12 hours (4) post-infection. Ureaplasmas were labeled with Vibrant Dil (in red, A), HEp-2 actin filaments stained with phalloidin-FITC (in green, B) and Hep-2 nuclei stained with TO-PRO-3 (in blue, C). In D, merging images A, B, and C. One minute after infection, ureaplasmas were observed inside HEp-2 cells, and after 30 minutes the presence of ureaplasmas inside cells increased. After 3, 8 and 12 hours of infection, ureaplasmas were observed throughout cells cytoplasm.

The images of infected cells were from the apical surface to the basolateral region and differentiated the actin filaments in green, from the blue luminescence of nuclei. Therefore the ureaplasmas were detected in red luminescence, discriminating their arrangements in the serial sections of the infected cells. The Dil solution did not show ureaplasmal cytotoxicity (data not presented) and allowed for differentiating the Hep-2 cells from ureaplasmal arrangements. Non-infected Hep-2 cells did not exhibit distinct intracellular Dil fluorescence. The images obtained showed adhesion and invasion of *U. diversum *in Hep-2 cells (figure 1). After one minute of infection, a few ureaplasmal cells were detected scattered and inside the Hep-2 cells (figure 1.1). After 30 minutes of infection, the ureaplasma persists, scattered, but increased inside the Hep-2 cells (Figure 1.2). At 3, 8 and 12 hours of infection, the microorganisms were detected mostly surrounding the perinuclear regions (Figure 1.3 and 1.4). The studied microorganisms showed no differences in their distribution when adhered to or inside the cytoplasm after 12 hours of infection. Ureaplasmal infection produced no cytopathic effects in Hep-2 cells in the studied period.

### Disposal of *U. diversum *in the infected HEp-2 cells

**Figure 2:**
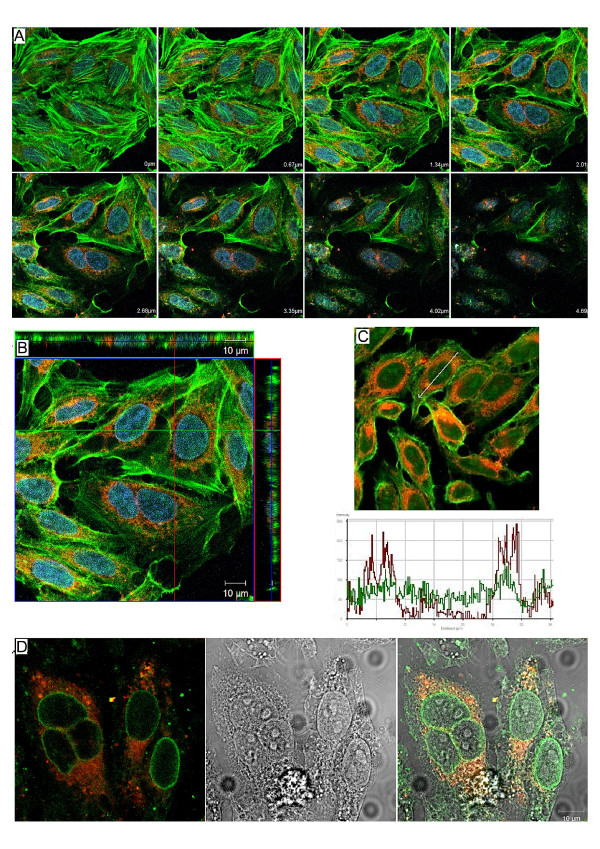
**Distribution of *U. diversum *in infected HEp-2 cells**. LSCM images showing the internalization of *U. diversum *in HEp-2 cells. Ureaplasmas stained by Dil (in red), actin filaments stained by phalloidin-FITC (green) and cells nuclei stained by TO-PRO-3 (in blue). **A and B: **Z-series of optical slices (A) and orthogonal projection (B) showing the presence and distribution of ureaplasmas inside HEp-2 cell. **C: **Image and graphic representation of HEp-2 cells after 12 hours post-infection. The arrow in confocal image indicates the cell in which the ureaplasma (in red) and actin (in green) was analyzed, and the detection of actin and ureaplasmas throughout this cell is represented in the graphic. **D: **Infected HEp-2 cells submitted to immunofluorescence with anti-lamin antibody (in green), showing ureaplasmas (in red) in the perinuclear region, but not inside the cell nuclei. All the images show ureaplasmas distributed throughout the HEp-2 cytoplasms, and concentrated in the perinuclear region, surrounding the nuclei.

Figure 2 shows disposition of ureaplasma in the studied infection. In figure 2A, optical slices from basal to apical regions of cells, including sections with the nucleus in the plane of the focus were also obtained. The ureaplasmas were detected in different sections of the Hep-2 cell cytoplasm but not inside the nucleus. The orthogonal sections after 3 hours of infection showed a red fluorescence from apical to basolateral regions and throughout the cytoplasm and perinuclear spaces. In figure 2B, images of the tri-dimensional distribution of Hep-2 cells three hours after infection were focused. As shown in figure 2A, red fluorescence was detected throughout the cytoplasm and perinuclear spaces.

Figure 2C is the graphic representation obtained with the software Imaris 3.1.3 (Bitplane AG) that confirms the perinuclear localization of ureaplasma. In addition, the Hep-2 cells were treated with RNAase for 30 min in all periods of infection and incubated with the goat anti-lamin antibodies (diluted 1:800 overnight) washed and exposed for 3 hours to anti-goat immunoglobulin (anti-goat FITC, diluted 1:100). The ureaplasma could be observed close to the nuclear lamin (Figure 2D); however, intranuclear ureaplasmas were not confirmed. The nuclear envelope lamina is a supramolecular protein assembly associated with the nucleoplasmic surface of the inner nuclear membrane. This delimitation was important to determine the presence of ureaplasmas in the perinuclear regions, but not inside the cell nuclei.

### Gentamicin invasion assay

The UB medium promoted the growth of studied ureaplasmas. The exposure of inoculum size of ureaplasmas used for gentamicin allowed no recovery in UB medium. However the ureaplasma of infected Hep-2 cells incubated with gentamicin and trypsinized allowed recovery of this microorganism. In this assay, it was possible to determine that the clinical isolates of ureaplasma revealed to be more concentrated in Hep-2 cells than reference strains. This quantification was determined by 10-fold dilutions of ureaplasma obtained after gentamicin assay in UB medium and expressed as Changing Color Units/ml (CCU/ml). Therefore, the internalization of studied ureaplasma in Hep-2 was confirmed and quantified in this assay. Gentamycin is impermeable to mammalian cells in the concentration used: it kills only the extra cellular ureaplasma but not the internalized bacteria. The rates of invasion were expressed as the percentage of CCU obtained after antibiotic exposure relative to the initial inoculum (frequency of invasion). The calculated p-value < 2.2e-16, test for equality of proportions with continuity correction, R project, Vienna, Austria allow for concluding that approximately 1% of the initial inoculum had survived the gentamicin treatment in type-strains and about 10% in clinical isolates. The ATCC strain has a high passage in UB medium. No differences were observed in frequency of invasion between high and low passages clinical isolates (p-value < 2.2e-16).

### Phospholipase C activity

**Figure 3:**
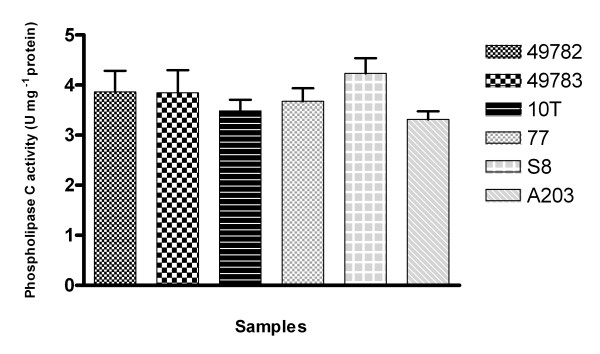
**Phospholipase C measured in *Ureplasma diversum *strains studied**. The absorbant was measured at 405 nm after incubation or 24 hours 37°C in UB broth with pNPPC.

The ureaplasmas were initially cultured at 37°C for 24 hours in one ml of UB broth with pNPPC. The supernatants were evaluated at a wavelength of 405 nm (OD_405_) in a Multiskan Microplate Reader (Flow Laboratories, Mississauga, Ontario, Canada). The phospholipase C activity was found in the studied ureaplasma and all produced high levels of this enzyme. The average activity was 2,476 to 3,396 pNPPC hydrolysis (U mg^-1 ^protein) (figure 3). This was the highest level that allowed detection of this compound in the present study. The phospholipase C activity also measured in sonicated ureaplasmas cells. The average activity was 0,783 to 0,821 pNPPC hydrolysis (U mg^-1 ^protein). These results showed that most activity is related to secreted enzyme. No differences were detected between the reference strains and clinical isolates. However this activity could be associated with a feature for invasion of ureaplasma.

## Discussion

Adhesion and invasion has been studied in a few mollicutes, most being human-originated species. Adhesion is considered an important feature to pathogenesis of these bacteria, and the invasion, a subsequent event, has been described in phagocytic or non phagocytic cells. Therefore chronic and recurrent mycoplasmosis may be explained in part by the reported failures of antibiotic treatments and immune response escape [3]. Vancini & Benchimol [13] reported *M. hominis *invasion in *Trichomonas vaginalis *and escaped from the vacuolization of trichomonad cytosol. This finding adds to understanding the challenging features of mollicute biology and their transmission among the hosts.

Consistent with other studied mollicutes, the infection described herein with *U. diversum *in Hep-2 cells allowed for identifying this ureaplasma as another mammalian cell invader and may also explain and support prior findings on some ureaplasmal infections in bovines.

CSLM has been used to detect mollicute invasion in non phogocytic cells confirming its advantage in detecting *U. diversum *invasion. The gentamicin invasion assay also confirmed this finding.

*U. diversum *was detected in Hep-2 cells one minute after infection. *M. penetrans *has been observed as early as 20 minutes after infection in HeLa cells [14], while in HEp-2 cells, the invasion occurred after 2 hours of infection [4]. Cell internalization after 20 minutes was also detected for *M. genitalium *in HeLa cells, and the mycoplasmas remained inside the cells for 7 days [15]. Winner *et al*. [9] observed penetration of *M. gallisepticum *in HeLa-229 and CEF cells occurred as early as five minutes after infection, and the intracellular mycoplasmas increased after 2 hours.

Ureaplasmas have not been previously reported as cell invaders and have never been compared in their invasion rate. In the present study, *U. diversum *showed a hasty invasion in Hep-2 cells.

Mollicute reference strains and the clinical isolates showed that these bacteria may have differences in growth and behavior when inoculated in animals or cell cultures [9,16]. The high passage strains have been described as more adapted to axenic growth in contrast to the low passage clinical isolates that have shown to be more aggressive in experimental infections [17]. Even in erythrocytes, HeLa-229 and CEF cells *M. gallisepticum *R low strain exhibited the highest invasion frequencies than the high passage strain [9,17]. The authors suggested a loss or switching off of the genetic information in this species for the invasion process in the high passage strains.

In the present study, we observed the same frequency of invasion in the gentamicin invasion assay for the high and low passages clinical strains.

Even when a field isolates, the higher passage ureaplasma may not lose or change yet the genetic expression for the studied invasion. In fact these mollicutes are few studied and quite different, therefore, they may reveal additional features for these bacteria.  

Buim (unpublished data) observed that the high (WVU 1853) and low passage isolates (MS1 and MS2) of *M. synoviae *also showed similar adhesion and invasion into Hep-2 cells and similarly surrounded the nucleus. Ueno *et al*. [18] observed the same results with high and low passages of *M. genitallium *infecting HeLa and endometrial human cells.

In this study, both ureaplasma reference strains and clinical isolates were detected inside the cells similarly surrounding the perinuclear regions but not inside the nucleus. The perinuclear arrangement was observed in other mollicutes [9,15,16]. Nevertheless, Ueno *et al*. [18] detected *M. genitalium *inside the nucleus after 30 minutes infection. Meseguer *et al*. [19] observed abnormal fluorescence in nuclear images in infected cultures, but failed to confirm the location of *M. pneumonie*.

The invasion of mollicutes is not completely established and different mechanisms have been proposed based on the studied mollicute and infected cells. Yavlovich et al. [20,21] showed the dependence of plasminogen-Pg in the invasion process of *M. fermentans MF*. The Pg treated MF were able to invade HeLa cells in three hours, but not the untreated MF.

The phospholipase C (PLC) is detected in many walled bacteria and is considered a virulence factor for tissue damage. In some mollicutes, PLC was detected [22] and associated with the cell invasion due to membrane and cytoskeleton modification. The mycoplasmal PLC was also associated with a host cell signal transduction cascade and the rearrangement of host cytoskeletal components [2,22].

The invading mycoplasmas generate uptake signals that trigger the assembly of highly organized cytoskeletal structures in the host cells. The invasion of *M. penetrans *is associated with tyrosine phosphorylation of a 145-kDa host cell protein that activate PLC to generate two additional messengers: phosphatidylinositol metabolites and diacylglycerol [23]. These observations support the hypothesis that *M. penetrans *use phospholipase to cleave membrane phospholipids, thereby initiating the signal transduction cascade. Moreover, the PLC appears to play a role in the escape from the primary vacuole and in gaining access to the cytoplasm [24]. *Listeria monocytogenes *deficient in PLC are 500-fold less virulent in mice [25].

The studied ureaplasma showed a high PLC activity, without differences between the reference strains and the clinical isolates. This activity explains similar behavior in Hep-2 cells and suggests the role of PLC as a factor for invasion of ureaplasma.

## Conclusions

The biological consequences of mycoplasma invasion are not established. Mycoplasma infections are quite variable and the diversity of species and hosts potentiate the complexity of this context. The intracellular location for these bacteria appears to be a comfortable niche for growth, allowing them to be more aggressive and more protected against immune response and antibiotics. Although *U. diversum *is a little studied species, its intracellular location adds this important feature to the understanding of mollicutes and explains their importance in bovine diseases.

## Methods

### *Ureaplasma diversum *and cell lines

Four isolates of *U. diversum *and two type-strains, ATCC 49782 and 49783, were studied. Isolates 77 and A203 were recovered from the vaginal mucus of a bovine vulvovaginitis (high passage), and the isolates 10T and S8 recovered from frozen bovine semen previously mixed with antibiotics in an artificial insemination center in Brazil (low passage). The isolates were initially identified with culturing characteristics and specie-specific PCR [26].

The Hep-2 (ATCC-USA CCL-23) cell lines were hosts to ureaplasmas in the present study and were previously certified to be free of mycoplasma by culture and PCR [27]. The cells were cultured in 5% of CO_2 _at 37°C in Minimum Essential Medium (MEM) containing 2 mM L-glutamine and Earl's balanced salts, supplemented with 10% fetal calf serum Cult Lab, São Paulo, Brazil). Twenty-four hours prior to mycoplasma infection, Hep-2 cell monolayers were established for 10-20% confluence on 13 mm glass slides, in 24-well micro plates (TPP - Switzerland), with one ml of MEM medium (Cult Lab, São Paulo, Brazil) for analysis by confocal microscopy. The Hep-2 cells used in the present study were analyzed for presence of mycoplasmas by culture and PCR.

### Labeling Mycoplasma cells

The methodology was based on Basemam *et al*. [28]. The ureaplasmas were first cultured in 2 ml of ureaplasma medium (UB) at 37°C and expanded to 50 ml in the same broth. In a logarithmic growth phase (based in colorimetric changes), the culture was centrifuged at 20,600 g for 30 minutes at 25°C. The pellets were homogenized by washing twice with PBS and incubated with carbocyanine dye solution (Vybrant™ Dil cell-labeling solution-Dil, V-22885, Molecular Probe, Eugene, Oregon, USA). Two-hundred microliters of Vibrant Dil (diluted at 1:200) were added to 10^5 ^- 10^7 ^mycoplasma cells in one ml of PBS and incubated at 37°C, for 40 minutes. The number of ureaplasma cells was determined by 10-fold dilution in UB medium and expressed as Changing Color Units/ml (CCU/ml). The labeled bacteria were centrifuged for 10 minutes at 20,600 g, at 25°C, washed twice with PBS, gently homogenized and transferred to the monolayer of Hep-2 cells.

### Inoculation of ureaplasma on Hep-2 cells [28]

The Hep-2 cells at 60 to 70% confluence corresponding to approximately 10^6 ^cells/glass slide were selected for ureaplasmal infection. These cells were initially washed with PBS and inoculated with 10^5 ^to 10^7 ^of labeled mycoplasmas contained in one ml of MEM with 2% bovine fetal serum. The sets of inoculated cells were incubated at 37°C in 5% CO_2 _atmosphere for one and 30 minutes, 3, 8 and 12 hours. After each period of infection, the bacterial suspension was gently removed and each well with cell monolayer was washed three times with PBS. The infected Hep-2 cells were then fixed with 3.7% formaldehyde in PBS for 30 minutes at room temperature and washed three times with PBS and treated with 0.05% Triton X-100 for 10 minutes.

### Labeling Hep-2 cells

For cytoskeleton visualization of infected and non infected Hep-2 cells, these cells were stained for 30 minutes at 37°C with phalloidin associated with fluoresceine-isothiocyanate (Sigma) diluted at 1:200. This fluorochrome was removed with three washings of PBSA. Then, the cells were treated with RNAase (10 mg/ml) for 30 minutes. The nuclei were stained with TO-PRO-3 (Molecular Probes, dilution 1:500). The preparations of Hep-2 cells and mycoplasmas were mounted with antifading solution (Vecta Shield, Vector Laboratories, Burlingame, CA, USA) on histological slides. The cells were fixed with 3.7% formaldehyde, treated with 0.5% Triton X-100 (10 minutes), exposed to goat anti-B lamin antibody overnight and incubated for 3 hours with anti-goat immuglobulin (1:100, Sigma) conjugated with fluorescein. The cells were washed three times with PBSA and mounted with Vecta Shield on histological slides.

### Confocal Laser Scanning Microscopy

The infected and non-infected Hep-2 cells were observed under Confocal Laser Scanning Microscope - CLSM (Carl Zeiss LSM 510, Germany, equipped with Argon laser, 488 nm, and 2 helium/neon 543 nm wavelengths) to visualize the luminescence of fluochromes.

Twenty fields with 8 to 10 infected and non infected cells with ureaplasma in each cytological preparation from each period were examined. A series of optical slices from basal to apical regions of cells, including sections with the nucleus in the plane of the focus were also obtained, and images of the tri-dimensional distribution of intracellular labelled-microorganims were focused. Images of all preparations were documented.

### Gentamicin invasion assay

The gentamicin invasion assay was performed to determine the invasion rate of viable ureaplasma inside the eukaryotic cells according to the Yavlovich *et al *[29]. Previously, the ureplasmas strains used in this study were tested for susceptibility to gentaminin in the concentration utilized in this assay (400 μg/ml). All strains were inhibited by gentamicin. The amount of 10^4 ^Hep-2 cells per well were seeded in 24-well micro plates. After 24 hours of incubation at 37°C in 5% CO_2_, the cell cultures were inoculated with 10^5 ^to 10^7 ^ureaplasmas (CCU/ml). The infected cells were incubated for three hours, washed three times with PBS and incubated for an additional three hours in MEM (1 ml/well) containing 400 μg/ml gentamicin to eliminate the non internalized ureaplasmas. The antibiotic solution was removed and the infected cells were trypsinized and cultured in UB broth. The remaining ureaplasmas were enumerated by CCU methodology and performed in triplicate. These results were compared with the initial ureaplasmal suspensions.

### Phospholipase C activity

Twenty micromoles of P-nitrophenylphosphorylcholine - pNPPC (Sigma) were used as a substrate to detect the phospholipase C activity of ureaplasma. The method is based on the hydrolysis of pNPPC, with the release of the chromogen, p-nitrophenol (NP). The analysis was performed in 96-well microtiter plates (TPP - Switzerland). The ureaplasmas were initially cultured at 37°C for 24 hours in one ml of UB broth with pNPPC. The supernatants were transferred to 96-well microtiter plates and evaluated at a wavelength of 405 nm (OD_405_) in a Multiskan Microplate Reader (Flow Laboratories, Mississauga, Ontario, Canada). The adjusted OD_405 _values from each ureaplasmal pNPPC hydrolysis were subtracted from the negative control wells. The negative control was the UB broth and pNPPC without bacteria. All tests were done in triplicate.

## Authors' contributions

LMM, PMU, MB and JT: all tests realized in this study. BAC and GMMS: confocal analysis. RLN, MY, RCO, AMSG: bacteria isolation. TAM: performed cell culture. ACBJR: data analysis. All authors read and approved the final manuscript.
